# Influence of lighting on visual performance

**DOI:** 10.5935/0004-2749.2023-0257

**Published:** 2024-04-18

**Authors:** Silvana Rossi, Newton Kara-José, Eduardo M. Rocha, Newton Kara-Júnior

**Affiliations:** 1 Department of Ophthalmology, Faculty of Medicine, University of São Paulo, São Paulo, Brazil; 2 Department of Ophthalmology, Faculty of Medicine, University of Ribeirão Preto, University of São Paulo, Ribeirão Preto, SP, Brazil

**Keywords:** Visual performance, Lighting, Visual acuity

## Abstract

**Purpose:**

This review emphasizes the effect of light on visual efficiency, the impact of different lighting focuses, types of lighting, and their influence on vision and productivity. Light sources and standards are intriguing subjects for ophthalmologists. Guidelines regarding the level of lighting influence on visual activities can enhance visual performance.**Methods:** This article was developed based on literature reviews, with a bibliographic survey conducted in databases such as PubMed, MEDLINE, Web of Science, Embase, LILACS, and SciELO.

**Results:**

Provides recommendations for understanding information regarding the influence of lighting on visual performance.

**Conclusion:**

Proper workplace lighting is crucial for improving visual efficiency, safety, productivity, and worker health. Efficient workplace lighting should avoid light sources directed towards the worker’s face, prevent harmful glare, be more intense in the work area, and uniform in the rest of the room. Ophthalmologists should be knowledgeable about and provide guidance on correct lighting to ensure patient comfort and satisfaction with visual correction.

## INTRODUCTION

The sources and patterns of light are interesting subjects for ophthalmologists when reviewing patients. Guidance on the influence of lighting on visual activities and the avoidance of hostile lighting, which is a source of eye irritation and discomfort, improves visual performance.

### Light and the human being

Human beings have lived for thousands of years in environments with a single source of light, the sun^(1)^. Fire was the dominant source 120,000 years ago, and electric light was discovered only 140 years ago^(2)^. Human activities, which are divided into two distinct periods (day and night), are performed 24 h a day in environments with numerous light sources. This abundance of artificial lights affects the brain that is not adapted to multiple light stimuli and causes visual, psychological, sensory, and physical changes^(2-6)^.

The lack of adaptation to multiple light sources manifests as discomfort, headache, nausea, mental confusion, and even epileptic seizures^(7-13)^.

### Color vision

Light demonstrates qualitative (wavelength) and quantitative (intensity) aspects. The visible light spectrum includes electromagnetic radiations with a wavelength of 400 to 700 nm, which correspond to ultraviolet (UV) and infrared lights, respectively.

Colors are detected by the cones in our retina. Sensitivity to colors is determined by the varying absorbance of cone photopigments across a range of wavelengths (short or long) in the visual spectrum. Long-, medium-, and short-wavelength cones identify red, green, and blue colors, respectively. These cones are capable of distinguishing a range of colors as they bleach in the presence of primary colors (red, blue, and green); other colors are obtained by mixing the primary colors^(14,15)^.

Color is directly related to light intensity. Below the threshold of photopic vision, cones are not stimulated, and the outside world appears gray. In this case, the rods perceive whether an object is more luminous than another.

Light rays of different wavelengths have different luminosity potentials. Under scotopic conditions (low luminosity), the peak luminosity is 500 nm (blue and green). Therefore, in the dark, when comparing sources with the same intensity, blue and green light appear brighter. Under photopic conditions (high luminosity), the peak luminosity is 555 nm (yellow and red). Therefore, these colors are the most visible during the day. In road traffic lights, yellow and red are the warning colors. This is because in photopic conditions, where the contrast of a bright spot is lower than in scotopic conditions, they are the most obvious.

The color of an object depends on the frequency of the wave that it reflects. If the object reflects all the colors of the light spectrum, it is perceived as white. If it absorbs all the incident light rays, it appears black. If the object reflects red and absorbs all other colors, it is perceived as red.

Achromatopsia is the inability to recognize any color; the visible spectrum is perceived as a gray band of various intensities. Dyschromatopsia refers to any abnormality in color vision. In protanopia, deuteranopia, and tritanopia, the colors red, green, and blue, respectively, are not perceived.

### Chromatic aberration

**Figure 1:**
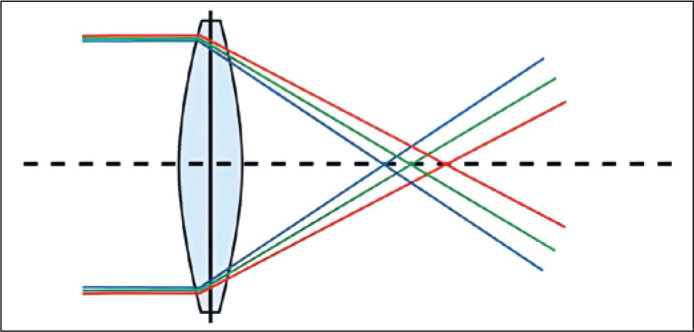
Chromatic aberration.

A beam of white light contains all the colors of the visible spectrum. Each color has a different wavelength and undergoes refraction differently. In the emmetropic eye, lights with a short-wavelength (blue) undergo greater refraction than lights with a longer wavelength (red). Furthermore, short-wavelength light focuses in front of the retina, and long-wavelength light focuses behind the retina (Figure 1). This is why glasses with yellow lenses improve the sharpness of an image because they eliminate the blue light. Additionally, they reduce the circle of least confusion; the luminous segments focused behind the retina can be approximated through visual accommodation.

**Figure 2:**
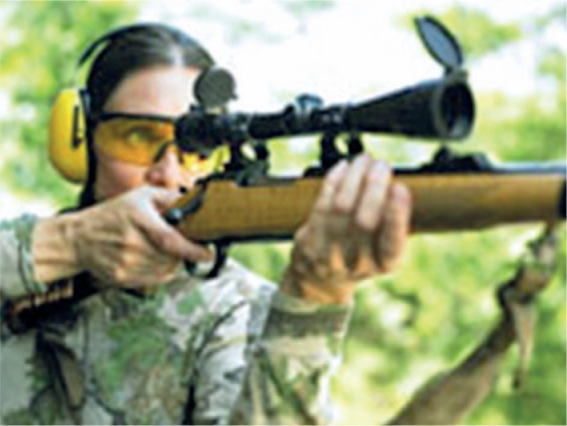
Shooters use yellow glasses to see distant targets better.

**Figure 3:**
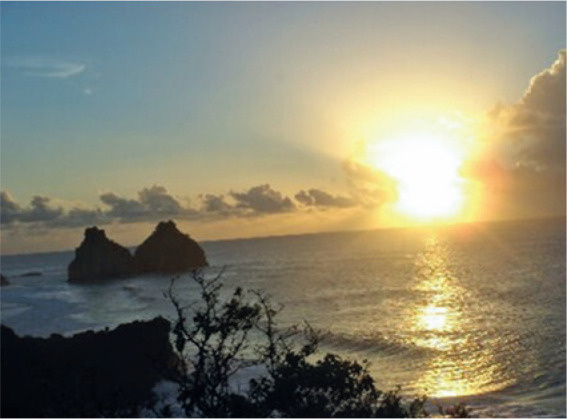
With the sun close to the horizon, the rays are filtered by atmospheric gases.

Shooters try to use yellow glasses to see distant targets better (Figure 2). Another indication for glasses with yellow lenses is driving a car at dusk. At dusk, the sun is close to the horizon, and its rays are filtered by atmospheric gases close to the earth’s surface (Figure 3). These atmospheric gases filter out colors with longer wavelengths, leaving behind more blue light which blurs vision.

**Figure 4:**
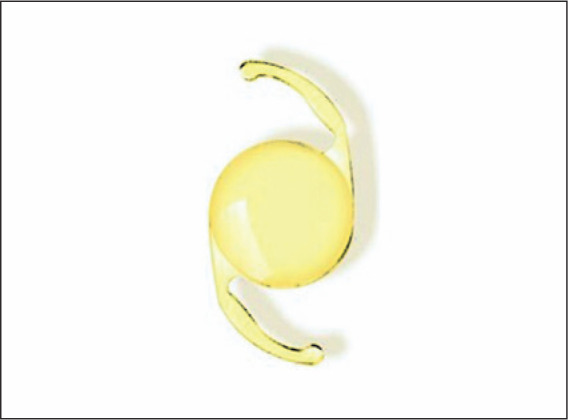
Yellow intraocular lens.

Since 2006, Kara-Junior et al.^(16)^ at USP have been studying the application of yellow intraocular lenses (IOLs) (Figure 4), designed primarily to protect the fovea from UV radiation and improve visual quality at dusk. Our studies have demonstrated a similar finding of improvement in visual quality in participants who underwent cataract surgery with a yellow IOL implantation. We also observed the influence of the IOL color on visual exams involving contrast sensitivity, such as the “blue/yellow” computerized perimetry and frequency doubling technology perimetry. However, during the 5-year follow-up, there was no evidence of its protective potential against retinal degenerative diseases^(16-18)^.

### White light

White light can influence vision in the following ways:

1. Better detail perception: White light improves contrast perception. It is useful in environments that expose the details of the tasks being performed, such as reading and manual work.
2. Photophobia: White light, when intense, causes glare and visual discomfort, especially in environments with bright floors and walls or when the light is directly focused into the eyes.
3. Influence on the circadian rhythm: White light influences the circadian rhythm, which regulates the body’s sleep-wake cycle, and causes insomnia. Exposure to white light at night can interfere with the production of melatonin, a hormone that regulates sleep. Thus, exposure to white light before going to sleep should be avoided. This also explains why relaxation environments have yellow lights^(12,13)^.
4. Eyestrain: Prolonged exposure to bright white light, especially on computer screens, smartphones, and other electronic devices, can cause eyestrain. This leads to symptoms such as dry eye, eye irritation, blurry vision, and headaches. Thus, it is important to take regular breaks and adjust screen brightness.

### Types of lamps

#### 1 - Incandescent lamp

In incandescent lamps, light is produced by heating a tungsten filament. It provides continuous, uniform, and comfortable lighting for visual efforts and emits a yellowish light, which is considered “warm light”^(3)^. However, incandescent lamps expend 95% of their energy on heat production and only 5% on lighting. Moreover, it reaches temperatures of up to 200°C, which can cause accidents, and has a short shelf life. Thus, although it is the most comfortable lighting for prolonged work, it has been banned in Brazil since 2016.

#### 2 - Fluorescent lamps

Fluorescent lamps are discharge lamps that produce white light as an electric current passes through a gas or vapor and ionizes it. They include mercury-vapor, sodium-vapor, and metal-halide lamps that are used in public lighting and large commercial spaces. Fluorescent lamps are more efficient in terms of energy consumption, as they expend only 30% of their energy on heat pro-duction and reach a maximum temperature of 45°C. They are considered a cold source of light.

#### 3 - Dichroic lamps

They are low voltage halogen lamps used in exhibition environments.

#### 4 - Halogen lamps

These lamps are similar to incandescent ones, which use halogen gas to prolong the lifespan of the tungsten filament. Although halogen lamps produce bright white light, they are not as energy efficient as incandescent lamps.

#### 5 - Light emitting diode lamps

Light emitting diode (LED) lamps use LEDs to produce illumination. They are very energy efficient and have a longer lifespan than incandescent and fluorescent light bulbs. LED bulbs can produce various colors and shades of light.

LED lamps expend only 5% of their energy on heat production and 95% on lighting. It is environmental impact is minimal because it does not contain toxic substances. Moreover, it reduces the risk of shock and burns because it runs on a very low voltage. Furthermore, LED lamps attract fewer insects and light up instantly without the need for ballast.

The lifespan of LED bulbs is up to 25 times longer than that of incandescent bulbs and three times longer than that of fluorescent bulbs. When used 8 h a day, LED bulbs can last for 17 years.

The LED lamp uses 50% less energy than a fluorescent lamp and 80% less energy than an incandescent lamp. Thus, it is considered the lamp of the future.

### Ideal lighting for every activity

Choosing the right light source depends on individual needs and preferences and the environment in which it will be used. The incandescent lamp, which generates continuous light, is more comfortable for performing detailing work than the fluorescent lamp, which produces flickering light. The following are factors to be considered:

1. *Intensity:* Bright lighting is required in work areas, whereas soft lighting is preferred in resting environments. Insufficient lighting can cause eyestrain and lack of focus.
2. *Color temperature:* The color temperature of the lighting affects the perception of the environment. Warm colors (yellow) create a relaxing environment, whereas cool colors (white) are used for activities that require attention and concentration.
3. *Even distribution:* Lighting should be evenly distributed throughout the room. Shadowy or overly bright areas that cause the eyes to adjust should be avoided.
4. *Absence of glare:* Lighting should not cause glare. It should not be too bright to make hinder vision.
5. *Energy efficiency:* Ideal lighting should be efficient in terms of energy consumption. Low-consumption light bulbs or more efficient technologies, such as LEDs, should be used.
6. *Reflections and glare:* Shiny surfaces, such as glass tables, or very light colors can reflect light excessively, thereby impeding clear vision.

Natural light is the best option whenever possible. It provides a sense of well-being and regulates the circadian rhythm, thereby improving sleep and mood during the day. When natural light is insufficient, it is important to invest in quality artificial lighting. LED lamps are an efficient and economical option that provide lighting close to that of natural light.

Inadequate lighting can cause headaches, eye irritation, decreased concentration, and decreased productivity.

The following are recommendations for making the best use of lighting:

1.Use lamps with flexible arms to direct light where it is needed.
2.Light should be directed in a way that prevents annoying shadows and glare.
3.The effects of different directions of lighting are as follows:
  - a.Light directed from behind an object helps distinguish it from its background.
  - b.Light directed from an upper angle reveals the shape and surface texture of an object.
  - c.Light directed from the front may reveal surface markings of an object. However, the ability to see texture is reduced^(3,6)^.

### Auxiliary light focus

The amount of light reaching the retina at 50 and 80 years of age is 50% and 20%, respectively, of that in a young person at 15 years of age^(19)^. Therefore, older adults typically need more lighting and/or additional focus in working environments. Supplementary lighting is also useful for people with decreased vision, nuclear cataracts, or presbyopia, as well those working with small objects^(6,19)^. In contrast, patients with posterior subcapsular cataracts may have better vision with less lighting^(19)^.

The luminaire support of lamps should be flexible (gooseneck type) to allow the user to adjust the distance and incidence angle of light. Illuminance is proportional to the distance between the lamp and the focused area, and the incidence of light regulates the direction of reflections.

Light should be directed from the left in right-handers and from the right in left-handers. This prevents the hand from creating a shadow in the workplace.

The light should be focused in the space between the eyes and the working area. If positioned anterior to the glasses, it can cause reflections on the back of the lens that are directed toward the eyes and causes discomfort and/or a ghost image (diplopia).

### Features of efficient lighting

Proper lighting can increase productivity by 10%-50% and decrease misreads by 30%-60%^(2)^. Ideally, the luminance should be intense and uniform in the work area and decrease in the underlying fields^(4-6)^ to avoid variation in pupil size and fatigue^(2,4-6)^. The surface of the workbench must be nonreflective (matte or dark coating), and for glass, metal, or light shades should be avoided^(2-5)^. Light should not be focused directly on the eyes to minimize the occurrence of glares, shadows, and reflections^(3-5,20-24)^.

### Effects of inadequate lighting

Inadequate lighting can cause visual disturbances, alteration of the blinking rhythm, dry eyes, tension headaches due to wrinkling of the facial muscles (mainly the frontal muscles), hyperemia of the palpebral border and conjunctiva, fatigue, nausea, irritation, cervicalgia, discouragement, loss of focus, and loss of attention^(8,9,11,25-27)^.

### Eye changes due to excess light

Excess sunlight containing UV radiation can damage the ocular surface and retinal photoreceptors. Light reflections from surfaces such as snow, water, and electric arcs can cause keratitis and conjunctival hyperemia^(25,26)^.

Intense or accumulated exposure to UV radiation is a risk factor for maculopathies, such as age-related macular degeneration^(28)^. Prolonged exposure to sunlight can cause a significant decrease in nocturnal visual acuity for up to two days. This phenomenon is particularly dangerous when driving vehicles at night^(20,23,24,28)^. However, moderate exposure to UV ra-dia-tion is a protective factor against the progression of myopia in children^(29)^.

### Protection from excessive light

#### 1 - Light from above

Human beings instinctively protect themselves using the following:

- Shields such as a turban, hat, cap, or open hand above the eye line.
- Glasses with absorbent or gradient lenses.
- Contraction of the frontal muscle with reduction of the palpebral fissure.
- Natural protectors such as a prominent orbit, eyebrows, pupillary miosis, and photophobia.

#### 2 - Light from the side

The following can be used for protection:

- Glasses with curved frames or side protection.
- Polarized lenses.

#### 3 - Light from the front

Light from the front can produce visual glare mainly in the presence of ocular opacifications, such as in cataracts with a posterior subcapsular component^(3,23,24)^. Frontal light can be minimized or avoided by wearing glasses with filtering lenses.

#### 4 - Color filters

- The gray filter almost completely blocks the sun’s luminosity.
- Brown lenses on sunny days increase the contrast and depth perception.
- Yellow lenses increase the contrast in a dimly lit environment; thus, they are indicated for night activities.
- Amber lenses increase stereopsis.
- Red lenses increase the contrast at dawn, dusk, and during snow^(20)^.

### Dispersion (diffraction) of light

Light diffraction is a phenomenon that occurs when light passes through an opening or around an object and spreads in different directions. This happens because light is a wave. The light beam can be partially deflected by the cornea, lens, iris, and retina. Conditions such as cataracts and corneal opacities can create obstacles to the passage of light, causing glare. This becomes more evident when there is a large difference in light intensity within a visual field such as when driving at night toward the headlights of an oncoming car.

Even small “water holes” or a posterior subcapsular opacity in the lens, if located on the visual axis, can significantly compromise the quality of vision.

Light rays, when passing through the eye, are dispersed by particles located in the cornea, lens, or vitreous humor. Diffraction decreases the contrast sensitivity and quantity and quality of the visual stimulus that reaches the retina. These particles increase with age, making older adults more sensitive to lighting conditions^(20)^.

Diffraction also explains the visualization of “floaters” that are mainly experienced by older adults with myopia. Diffraction also explains the red-orange color of the sky during sunrise and sunset. During these times, the light crosses an area closer to the earth that contains more suspended refractive particles. On clear days, the bluish color of the sky can be attributed to the lower concentration of particles in that space^(1,23)^.

### Identification of the light stimulus

Light energy is quantified in “quanta.” Reportedly, 50 quanta of light falling on the cornea can generate a detectable signal. Factors that influence the detection of the light stimulus include the following:

- Dark adaptation: In the dark, the eye becomes progressively more sensitive to light stimulation and reaches a maximum in 30 min. Therefore, under scotopic conditions, the eye is able to perceive lower intensities of light.
- Contrast: The greater the difference between the brightness of the object and the background, the greater the ability to detect an object (e.g., a dark “floater” can be noticed looking at a white wall).

Opacification of the lens nucleus, which is commonly called a cataract, compromises contrast sensitivity the most. Because this type of opacity progresses slowly, if there is no opacification of the other layers of the lens, it can take decades for the contrast sensitivity to impact the visual acuity measured with the Snellen Chart. However, in the real world, a significant reduction in contrast sensitivity could occur before, impairing daily and professional activities, especially in low-light environments.

**Figure 5:**
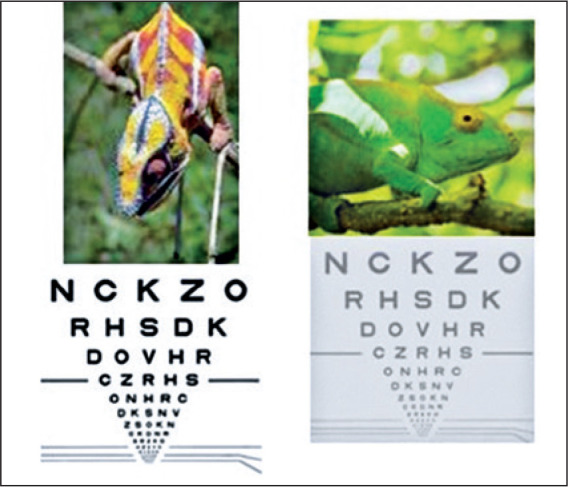
Contrast sensitivity.

Contrast is one of the main components of visual potential. Contrast sensitivity allows us to distinguish between colors and objects, especially in low-light conditions. In the real world, it is responsible for the vision of a black hole on a gray sidewalk in the dark (Figure 5).

During cataract surgery, aspherical IOLs can be implanted to enhance contrast sensitivity in low-light environments^(30-33)^. Bior trifocal IOLs can also be implanted for the same purpose. Although they reduce contrast sensitivity, they decrease dependence on near optical correction^(34-39)^.

To enhance the visual acuity of patients, ophthalmologists should educate them on proper lighting in addition to prescribing the best optical correction for their eye defects.
